# Reductive stress and the role of antioxidants in male infertility: a narrative review

**DOI:** 10.1007/s00404-025-08184-3

**Published:** 2025-09-17

**Authors:** Efthalia Moustakli, Panagiotis Christopoulos, Anastasios Potiris, Athanasios Zikopoulos, Alkis Matsas, Ioannis Arkoulis, Despoina Mavrogianni, Eirini Drakaki, Athanasios Zachariou, Peter Drakakis, Sofoklis Stavros

**Affiliations:** 1 Laboratory of Medical Genetics, Faculty of Medicine, School of Health Sciences, University of Ioannina, 451 10 Ioannina, Greece; 2 Second Department of Obstetrics and Gynecology, Aretaieion University Hospital, Medical School, National and Kapodistrian University of Athens, 115 28 Athens, Greece; 3 Third Department of Obstetrics and Gynecology, University General Hospital “ATTIKON”, Medical School, National and Kapodistrian University of Athens, Rimini 1, 124 62 Athens, Greece; 4 Laboratory of Experimental Surgery and Surgical Research “N.S. Christeas”, Medical School, National and Kapodistrian University of Athens, 115 27 Athens, Greece; 5 First Department of Obstetrics and Gynecology, Alexandra Hospital, Medical School, National and Kapodistrian University of Athens, 115 28 Athens, Greece; 6 Department of Urology, School of Medicine, Ioannina University, 451 10 Ioannina, Greece

**Keywords:** Reductive stress, Antioxidant, Male infertility, Seminal parameters, Redox equilibrium

## Abstract

**Background:**

The ability of antioxidant therapy to mitigate oxidative stress (OS)-induced sperm function impairment makes it a popular treatment for male infertility. Reductive stress (RS), a condition characterized by an overcompensation in redox balance that favors reduction over oxidation, may be brought on by excessive or extended antioxidant use, according to mounting evidence. The purpose of this review is to examine the processes by which an excess of antioxidants causes RS and to evaluate any potential negative impacts on men's reproductive health.

**Methods:**

A comprehensive overview of recent clinical and experimental studies focused on the effects of excessive antioxidant use on redox biology, mitochondrial function, spermatogenesis, and sperm quality.

**Results:**

RS has been shown to affect sperm growth and function, interfere with cellular signaling, and damage mitochondrial integrity. Antioxidants are commonly employed, although there are currently no clear clinical guidelines or biomarkers for diagnosis to monitor redox equilibrium.

**Conclusions:**

In male infertility treatments based on antioxidants, RS poses a paradoxical risk. A personalized, balanced approach to antioxidant therapy is essential, alongside the development of biomarkers and standardized protocols to ensure redox homeostasis and avoid potential harm.

## Introduction

Approximately 15% of couples worldwide experience infertility, with male factors accounting for nearly 50% of these cases [1]. OS has emerged as a crucial factor among the various causes affecting sperm function and male fertility potential. Lipid peroxidation, DNA damage, and protein oxidation within spermatozoa result from OS, which occurs due to an imbalance between endogenous antioxidant defenses and reactive oxygen species (ROS) generation [2, 3]. Due to their specialized structural and functional properties, spermatozoa are particularly prone to oxidative damage, which can impair their morphology, motility, and fertilization ability [4].

Antioxidant therapy, which aims to neutralize excessive ROS and restore redox balance, has been a widely used intervention in response [5]. In clinical and experimental settings, numerous plant-derived compounds, coenzyme Q10, vitamins C and E, and other common antioxidants have consistently demonstrated improvements in sperm parameters [6]. The premise behind these treatments is that increasing antioxidant levels will consistently increase male fertility. They are frequently used empirically [7].

However, recent research challenges this simplistic view by revealing that excessive or prolonged antioxidant supplementation can shift the cellular redox environment toward RS —a state marked by an overabundance of reducing equivalents, such as glutathione and NADPH [8, 9]. In addition to perturbing the finely regulated processes of spermatogenesis and sperm maturation, this reductive imbalance may compromise mitochondrial bioenergetics and disrupt key redox-sensitive signaling pathways. Ironically, cellular dysfunction and infertility may be linked to reduce RS, a phenomenon that is less comprehensively understood than OS [10, 11].

The concept of a ‘U-shaped curve’ in antioxidant therapy is often employed to describe this paradoxical phenomenon, whereby both insufficient and excessive antioxidant levels may exert harmful effects [12, 13]. It reinforces the fundamental importance of maintaining redox homeostasis. Although antioxidant therapy is increasingly employed in the treatment of male infertility, there is still an absence of validated biomarkers and standardized clinical protocols to monitor redox balance and minimize the risk of RS-related adverse effects [14].

The objective of this narrative review is to delineate the mechanisms driving RS and critically evaluate the consequences of excessive antioxidant supplementation on male reproductive function. The influence of lifestyle factors, particularly dietary patterns such as the Mediterranean diet (MD) and physical activity, on OS and male reproductive health is covered in this review in addition to therapeutic antioxidant supplementation. These elements use intricate antioxidant and anti-inflammatory processes to affect sperm function and systemic redox homeostasis [15]. By considering both lifestyle and therapeutic interventions, this review aims to provide an integrated perspective on current approaches to managing male infertility related to redox imbalance. Furthermore, it critically examines existing clinical and experimental evidence to highlight the potential risks and benefits of antioxidant therapy and advocates for a cautious, individualized approach in treatment strategies. This review's main goal is to merge and incorporate current paradigms into a coherent, therapeutically practical perspective, even though it does not propose a new conceptual framework. In addition to identifying priority areas for further research, it aims to improve the accessibility and usefulness of current knowledge for clinicians and researchers by combining biochemical, clinical, and lifestyle evidence into a cohesive narrative.

## The U-shaped curve of antioxidants: too much of a good thing

Antioxidant therapy in male infertility is based on the principle of neutralizing excess ROS to reestablish redox homeostasis and safeguard spermatozoa from oxidative damage [16]. Preserving a balanced redox environment is vital, given recent findings that antioxidant effectiveness follows a U-shaped dose–response relationship, where both deficient and excessive antioxidant levels may be harmful [17].

At one end of the spectrum, inadequate antioxidant defenses permit unchecked OS, leading to lipid peroxidation of sperm membranes, DNA fragmentation, and impaired sperm motility and viability. In this scenario, antioxidant supplementation can be beneficial by scavenging free radicals and preventing further cellular injury [18].

RS, a harmful but less-recognized condition compared to OS, results from an excessive accumulation of reducing agents, such as glutathione, thiols, and NADPH. This hyper-reducing state may compromise physiological redox signaling pathways that are essential for acrosome response, capacitation, and normal sperm maturation [19]. Moreover, diminished OS may disrupt mitochondrial activity, cause reduced ATP synthesis, and heighten apoptotic risk [20].

Complex and multidimensional mechanisms underlie the negative effects of RS. An excessive increase in reducing equivalents may modify the redox status of key cysteine residues on proteins, resulting in altered signaling cascades and enzyme functions [21]. ROS at physiological levels are critical for normal sperm function, serving as key signaling molecules; however, elevated levels of RS may interfere with their synthesis and impair sperm performance. Disruptions caused by this imbalance can lead to abnormal cellular processes that impair sperm quality and reduce their fertilizing ability [4, 22].

Clinically, the U-shaped curve illustrates the risks associated with excessive antioxidant supplementation in the absence of proper professional oversight and individualized patient assessment [23]. Despite the widespread application of antioxidant therapy, accurate assessment of redox status and the advancement of treatment protocols necessitate the establishment of standardized procedures and reliable biomarkers. To mitigate the adverse effects of RS, optimization of antioxidant therapy necessitates personalized approaches that account for baseline oxidative status, precise dosing, and treatment duration [24].

**Fig. 1:**
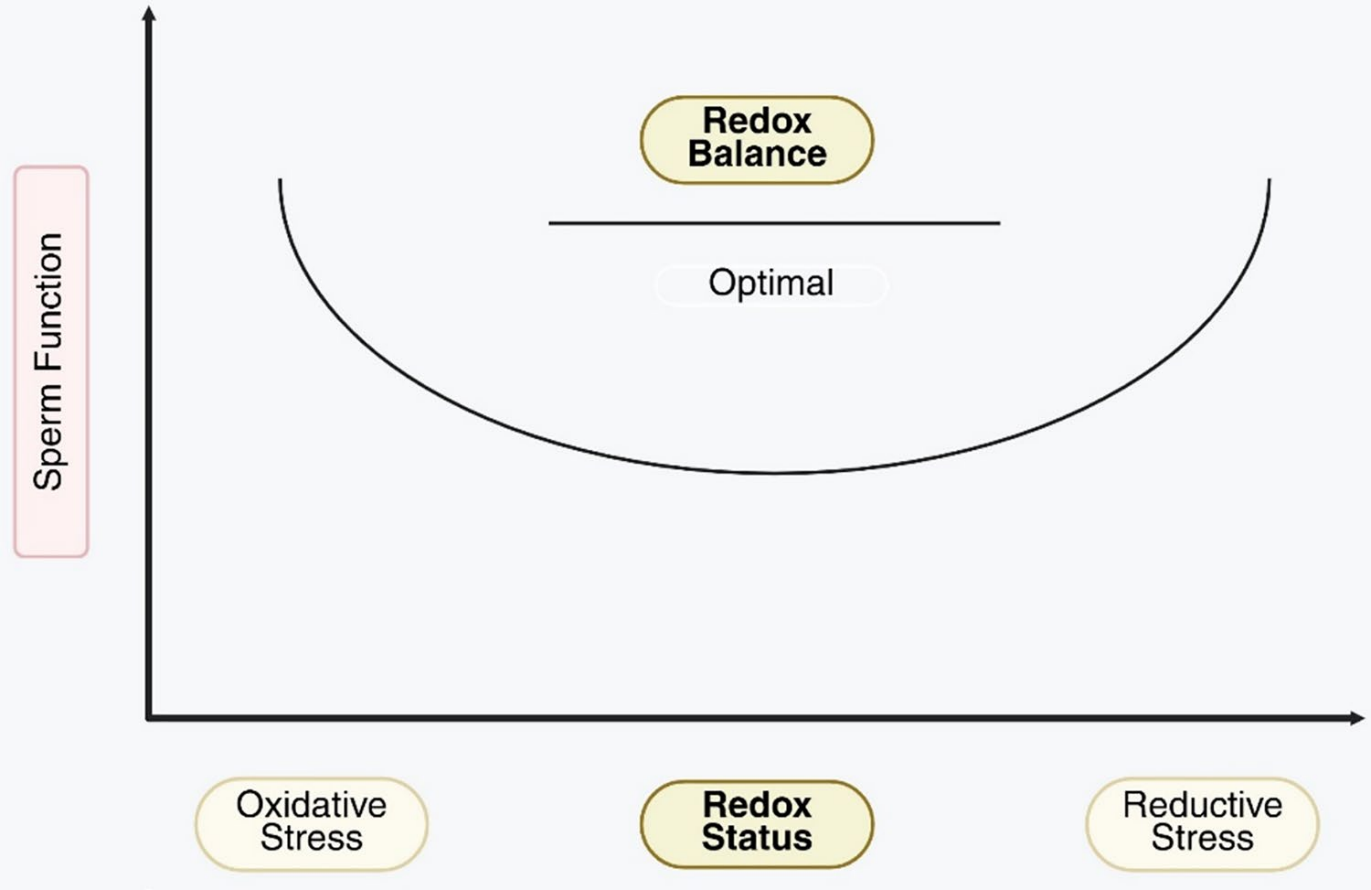
Illustration of the U-shaped relationship between redox status and sperm function

The paradigm of the U-shaped curve emphasizes that antioxidants are not always advantageous in all situations and at all levels. To avoid disrupting the balance from OS to RS, antioxidant therapy must be judiciously administered to achieve and sustain redox homeostasis, a critical factor in the management of male infertility [12] (Fig. 1).

Both excessive OS (left side of the curve) and excessive RS (right side of the curve) are associated with impaired sperm function. Optimal sperm function is achieved at a balanced redox state (center of the curve), where physiological levels of ROS are maintained for normal cellular signaling without causing oxidative damage.

## Mechanisms of RS in male reproduction

It is becoming more widely acknowledged that spermatozoa are early indicators of environmental and systemic redox imbalance. Sperm cells are particularly susceptible to oxidative and reductive imbalances because of their structural features, which include a high membrane concentration of polyunsaturated fatty acids, weak antioxidant defenses, and rapid metabolic activity [4]. The male reproductive system is among the first to react to endogenous and external stresses that disrupt redox homeostasis because of its sensitivity. Therefore, before symptoms appear in other tissues, sperm quality and function frequently reflect more extensive physiological disturbances [25]. An important window into the reproductive and systemic effects of redox dysregulation is provided by knowledge of how sperm biology is impacted by RS [22].

The delicate redox balance is disrupted when the intracellular reducing environment becomes excessively elevated, resulting in RS. A hyper-reductive state within the male reproductive system can impair multiple cellular and molecular processes essential for sperm production, maturation, and function [26].

### Disruption of redox signaling

Redox signaling, which governs various cellular functions such as gene expression, cell death, and metabolism, depends on the reversible oxidation and reduction of cysteine residues within proteins [27]. These cysteine residues can be overmodified by excess reducing agents like glutathione (GSH) and NADPH, resulting in abnormal protein activity and compromised signal transmission. Two crucial processes for fertilization, the acrosome reaction and capacitation in spermatozoa, are regulated by redox signaling. Therefore, by blocking these essential signaling pathways, RS can reduce sperm's ability to fertilize [28].

### Mitochondrial dysfunction

Sperm motility depends on the process of oxidative phosphorylation, which is how mitochondria produce ATP. Physiological levels of ROS, although generated as mitochondrial byproducts, function as signaling molecules essential for maintaining mitochondrial homeostasis [10]. RS may compromise mitochondrial function through interference with redox-sensitive enzymes and electron transport chain components, leading to reduced ATP generation and increased risk of apoptosis and mitochondrial permeability transition. This mitochondrial dysfunction leads to a reduction in sperm motility and viability [29, 30].

### Impairment of spermatogenesis

Redox-sensitive pathways precisely regulate the proliferative and differentiative phases of spermatogenesis, a highly coordinated process. RS, by altering the redox environment of the testes, can adversely affect Sertoli cell function as well as germ cell proliferation and differentiation. Excess reducing equivalents, for example, may impair sperm production by inhibiting the physiological ROS-mediated signaling essential for meiosis and spermiogenesis [31].

### Altered protein folding and degradation

Proteostasis mechanisms and redox-sensitive chaperones play a critical role in maintaining protein homeostasis within sperm cells [32]. An overly reducing environment may disrupt the formation of disulfide bonds required for correct protein folding, resulting in misfolded proteins and weakened structural integrity of sperm. Furthermore, RS may disrupt proteasomal degradation pathways, resulting in the accumulation of damaged proteins [33] **(**Table 1**)**.

**Table 1:** Potential mechanisms by which RS impacts sperm function

Mechanism	Description	Impact on sperm/function
Disruption of redox signaling [86]	Excess reductants modify cysteine residues, impairing redox-sensitive pathways	Impaired capacitation, acrosome reaction, fertilization
Mitochondrial dysfunction [87]	Reductive environment alters ETC and ROS signaling	↓ ATP production, ↑ apoptosis, ↓ motility
Impaired spermatogenesis [88]	Inhibits physiological ROS roles in meiosis and germ cell differentiation	Abnormal sperm production, defective morphology
Protein folding dysregulation [89]	Over-reduction impairs disulfide bond formation and proteostasis	Structural instability, accumulation of misfolded proteins

RS, reductive stress; ETC, electron transport chain; ROS, reactive oxygen species

## Antioxidant therapy hype vs. evidence

The strong connection between OS and sperm dysfunction has significantly contributed to the widespread adoption of antioxidant therapy in treating male infertility [34]. The extensive use of antioxidant supplements, commonly promoted due to their commercial availability and anecdotal reports of improved fertility, often occurs without thorough clinical assessment or personalized advice [35].

### The hype

Antioxidants are appealing, because they have the potential to counteract harmful ROS, which are known to break sperm DNA, promote lipid peroxidation, and reduce motility. Antioxidant supplements are widely believed to benefit male reproductive health. Consequently, many infertile men, sometimes without medical supervision, self-administer high doses of vitamins C and E, coenzyme Q10, and herbal extracts [36].

The assumption that higher intake confers greater benefits is reinforced by the portrayal of antioxidants as inherently "natural" and "safe," which facilitates prolonged use and administration of elevated doses [37]. Although findings are often inconsistent and sometimes contradicted by other studies, certain clinical trials have reported improvements in semen parameters following antioxidant treatment, fueling heightened enthusiasm [38].

### The evidence

Despite encouraging initial findings, there is currently little and conflicting clinical evidence to support the routine use of antioxidants in male infertility. The lack of uniform dosing schedules, varied patient groups, and small sample numbers in many trials makes it difficult to draw definitive conclusions from them [39].

Additionally, recent advances in redox biology have cast doubt on the oversimplified belief that antioxidants invariably exert beneficial effects [40]. High levels of antioxidant supplementation may alter redox homeostasis through the induction of RS, leading to impaired sperm function and disruption of normal physiological signaling. Nevertheless, antioxidant therapy lacks established protocols, and this topic is seldom covered in clinical guidelines [26].

### Balancing expectations

The gap between the widespread excitement about antioxidant therapy and the supporting data highlights the critical need for careful, evidence-based treatment strategies [41]. Safer and more effective alternatives to indiscriminate antioxidant supplementation may involve personalized assessment of redox status and targeted interventions. While antioxidant therapy holds promise, careful consideration of its indications, dosage, and potential risks is crucial to avoid inadvertently compromising male reproductive health [8, 24].

## Antioxidant therapies in male infertility

Antioxidant supplementation is among the most commonly used empirical treatments for male infertility related to OS. A wide variety of antioxidants are utilized clinically, either alone or in combination, with the goal of improving sperm quality and reproductive outcomes [42]. However, therapeutic regimens vary significantly, reflecting the absence of consensus on the optimal compounds, dosages, and duration of treatment.

Vitamin C and vitamin E are classic antioxidants frequently employed due to their ability to neutralize free radicals and protect sperm membranes from lipid peroxidation. Moderate dosing of these vitamins has been reported in some clinical studies to improve sperm motility and DNA integrity, although findings across studies remain inconsistent [43]. Coenzyme Q10 (CoQ10), a mitochondrial cofactor with potent antioxidant properties, has also gained attention for its role in supporting energy production and reducing oxidative damage; supplementation with CoQ10 has been linked to enhancements in sperm motility and concentration in certain infertile men [44]. Carnitine, important for the transport of fatty acids into mitochondria, exhibits antioxidant effects as well and is thought to improve sperm metabolism and motility, often being included in combination therapies [45]. N-acetylcysteine (NAC), a precursor to glutathione, increases intracellular antioxidant capacity and has shown potential in improving sperm parameters, though its excessive use carries the risk of inducing RS [46]. Additionally, plant-derived antioxidants, such as polyphenols and flavonoids, are increasingly utilized for their antioxidant and anti-inflammatory properties, yet clinical evidence supporting their efficacy remains limited [47, 48].

Traditional antioxidant therapy may benefit from a complementary approach from emerging dietary patterns like the MD, rich in polyphenols and flavonoids such as quercetin and kaempferol, which exhibit strong anti-inflammatory and antioxidant effects [49]. Preliminary studies suggest that adherence to the MD is linked to reduced oxidative damage in spermatozoa and improved semen quality, possibly due to enhanced mitochondrial efficiency and redox balance [4]. Incorporating naturally occurring antioxidants, such as flavonoids, into dietary recommendations may provide a more sustainable, safe, and synergistic approach to controlling OS in male infertility, despite the lack of scientific trials [50].

Combination antioxidant therapies are frequently favored by clinicians based on the hypothesis that synergistic effects may better restore redox balance and improve sperm function [39]. However, the diversity of combinations and dosing regimens complicates the interpretation of results and hinders the establishment of standardized treatment protocols [51]. Despite some promising outcomes, the lack of clear diagnostic criteria to identify OS and guide therapy continues to challenge clinical decision-making. Dosages are often empirically determined, and the potential risks of overdosing, including the onset of RS, are seldom considered [52] **(**Table 2**)**.

**Table 2:** Summary of common antioxidants used in male fertility support

Antioxidant	Proposed Mechanism	Reported Benefits	Typical Dosage	Limitations
Vitamin C [90]	• Scavenges ROS • Protects sperm DNA	Improved sperm motility & DNA integrity	500–1000 mg/day	Limited efficacy at high doses; may induce RS
Vitamin E [91]	• Prevents lipid peroxidation in sperm membranes	Improved membrane stability & motility	200–400 IU/day	Pro-oxidant effects at high doses
Coenzyme Q10 [92]	• Supports mitochondrial function and energy production	Improved sperm motility & concentration	100–300 mg/day	Variable response; long-term effects unclear
Carnitine [45]	• Enhances mitochondrial fatty acid transport	Increased motility & sperm metabolism	1–3 g/day	Often used in combination; evidence mixed
N-acetylcysteine (NAC) [93]	• Precursor to glutathione • Replenishes antioxidant stores	Improved DNA integrity & motility	600–1200 mg/day	Risk of RS with prolonged use
Polyphenols / Flavonoids [94]	• Plant-derived antioxidants • Anti-inflammatory properties	Potential anti-oxidative protection	Variable, depending on source	Limited clinical evidence; unpredictable bioavailability

**Fig. 2:**
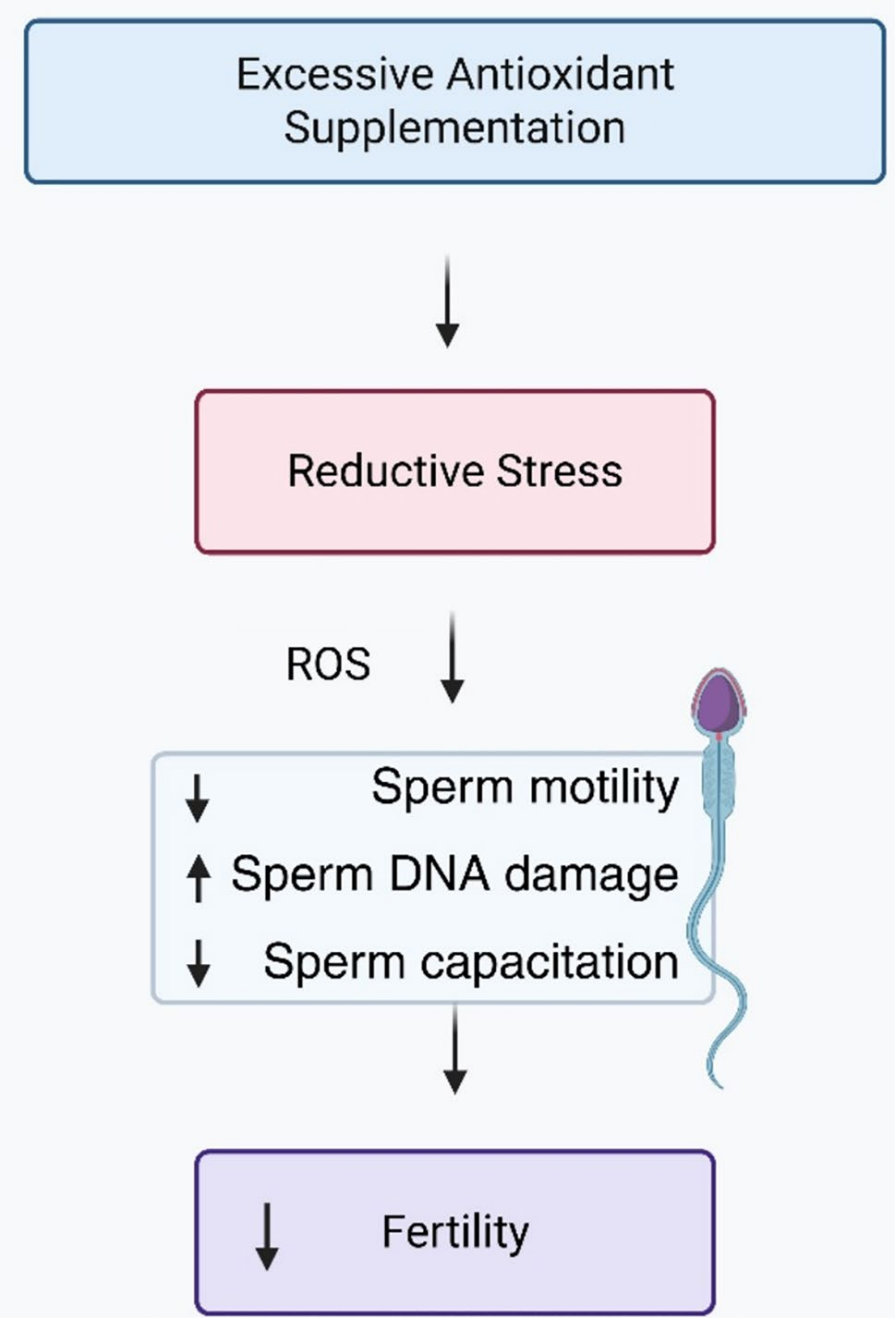
A conceptual overview of how excessive antioxidant supplementation may lead to RS and its potential impact on sperm function

There is a great deal of variation in study design, sample sizes, antioxidant kinds, dosages, and treatment durations, even though many clinical trials show improvements in sperm parameters after antioxidant supplementation. Drawing firm conclusions about efficacy is difficult due to this heterogeneity and common methodological flaws like small cohorts or a lack of placebo controls [39]. Studies that critically evaluate possible negative effects and take into consideration the variation in baseline redox state among individual patients are also scarce. To create standardized procedures and maximize customized antioxidant treatments, more carefully planned, extensive randomized-controlled trials are required. As a result, a cautious interpretation of the data is justified [17] (Fig. 2).

## Lifestyle interventions in male infertility

Lifestyle factors like PA and diet are important in regulating OS and affecting reproductive outcomes, in addition to nutritional therapies. Maintaining sperm quality requires regular moderate-intensity exercise, since it has been demonstrated to boost mitochondrial function, lower systemic inflammation, and strengthen endogenous antioxidant defense mechanisms [53, 54]. Conversely, excessive or high-intensity training may lead to oxidative damage in spermatozoa and increase the generation of ROS, particularly if there is insufficient recuperation. Balanced physical activity has been associated in numerous studies with better semen parameters, hormonal balance (e.g., testosterone levels), and male reproductive potential [55]. Therefore, implementing structured and personalized exercise routines may enhance systemic redox balance and serve as a valuable adjunct to antioxidant therapy [56].

Supplements and dietary patterns like the MD, which are high in flavonoids, polyphenols, and natural antioxidants, can work in concert to maximize mitochondrial function and redox homeostasis. Confounding variables that affect the efficacy of antioxidant treatments include individual differences in dietary habits, lifestyle choices, and baseline redox status. The necessity for comprehensive mechanistic investigations addressing these interactions is highlighted by this complexity, which may help to explain the uneven clinical outcomes seen [49].

Furthermore, many antioxidant treatments do not account for individual variability in baseline redox status or the underlying causes of infertility. This “one-size-fits-all” approach may contribute to inconsistent clinical results and underlines the need for personalized treatment strategies [57]. Moving forward, advances in male infertility management will depend on the development of reliable biomarkers and personalized medicine approaches that guide antioxidant therapy, maximizing benefits while minimizing risks [43, 58].

## Redox homeostasis & biomarkers: diagnostic challenges

Male fertility and proper sperm function depend on maintaining redox equilibrium, yet it is still very difficult to measure this balance precisely in clinical settings [59]. Given the complexity of redox biology, where both oxidative and reductive imbalances can be detrimental, accurate diagnostic methods are essential for monitoring the redox environment and guiding antioxidant therapy. Regretfully, standardized and reliable biomarkers for evaluating oxidative or RS in infertile men are currently lacking [60].

To quantify OS, several tests have been developed, such as measuring ROS, total antioxidant capacity, lipid peroxidation products, and sperm DNA fragmentation [61]. Although these tests offer valuable insights, they frequently fall short in terms of repeatability, sensitivity, or specificity. Additionally, they typically only depict one facet of the redox balance, missing the dynamic interaction between reductants and oxidants. Given that RS is less clearly defined and infrequently assessed in standard diagnostics, this constraint is especially pertinent when detecting it [62] **(**Table 3**)**.

**Table 3:** Redox-related biomarkers in the assessment of male infertility

Biomarker	Measures	Strengths	Limitations	Clinical use status
ROS assays (chemiluminescence) [95]	ROS levels	Direct ROS detection	Requires fresh samples; high variability	Research & limited clinic
Total Antioxidant Capacity (TAC) [96]	Overall antioxidant defense	Easy to perform	Lacks specificity; influenced by diet	Clinical/lab use
Lipid peroxidation (MDA,4-HNE) [97]	Oxidative damage to membranes	Indicator of oxidative damage	Late marker; not redox-specific	Mostly research
DNA fragmentation (TUNEL, SCSA) [98]	Oxidative damage to DNA	Correlates with infertility	Not specific to redox imbalance	Clinical use
Glutathione (GSH/GSSG ratio) [99]	Redox buffer status	Can indicate RS	Requires advanced lab capabilities	Experimental
Redox-sensitive probes (e.g., Nernst potential) [100]	Cellular redox potential	Highly sensitive, theoretical potential	Limited availability; not routine	Experimental

A variety of tests—including ROS assays, TAC, lipid peroxidation markers, and sperm DNA fragmentation analysis—are currently used to assess OS. However, none offer a comprehensive view of redox homeostasis [60]. Moreover, assays that can reliably detect RS are largely absent from clinical settings. Despite their potential, novel approaches like glutathione redox ratios (GSH/GSSG) and redox-sensitive biosensors are often overlooked owing to their complexity and expense [63]. For male infertility, the development of validated, standardized, and easily accessible biomarker panels is essential to enhancing diagnostic precision and facilitating tailored redox-based treatments [64].

Another challenge is that redox status fluctuates over time both between individuals and within the same patient, influenced by factors, such as nutrition, lifestyle, environmental exposures, and underlying medical conditions. Because of this diversity, it is challenging to establish global reference ranges and to determine precise intervention thresholds [65, 66].

Moreover, the lack of reliable biomarkers limits the capacity to customize antioxidant therapies effectively [67]. In the absence of reliable markers for baseline redox status and treatment response, clinicians must rely on empirical approaches, which increases the risk of both undertreatment and overtreatment. Therefore, excessive antioxidant intake may induce RS, potentially resulting in unforeseen detrimental effects on sperm function [12].

Comprehensive biomarker panels that can precisely measure redox homeostasis must be developed and validated immediately to improve the management of male infertility. Ideally, these tools should detect oxidative and reductive imbalances, yield consistent results, and be practical for use in clinical settings [68]. By enabling individualized antioxidant therapy tailored to the patient’s unique redox profile, the application of such diagnostic technologies can enhance both therapeutic efficacy and efficiency [69].

## Clinical implications

Recognizing RS as a potential adverse effect of antioxidant therapy has important therapeutic implications for the clinical management of male infertility [14]. Recent research indicates that, despite their widespread reputation as safe supplements with several health benefits, the excessive or unregulated use of antioxidants may have detrimental effects on sperm function [70]. This challenges the conventional treatment framework and demands a more sophisticated, tailored approach.

**Fig. 3:**
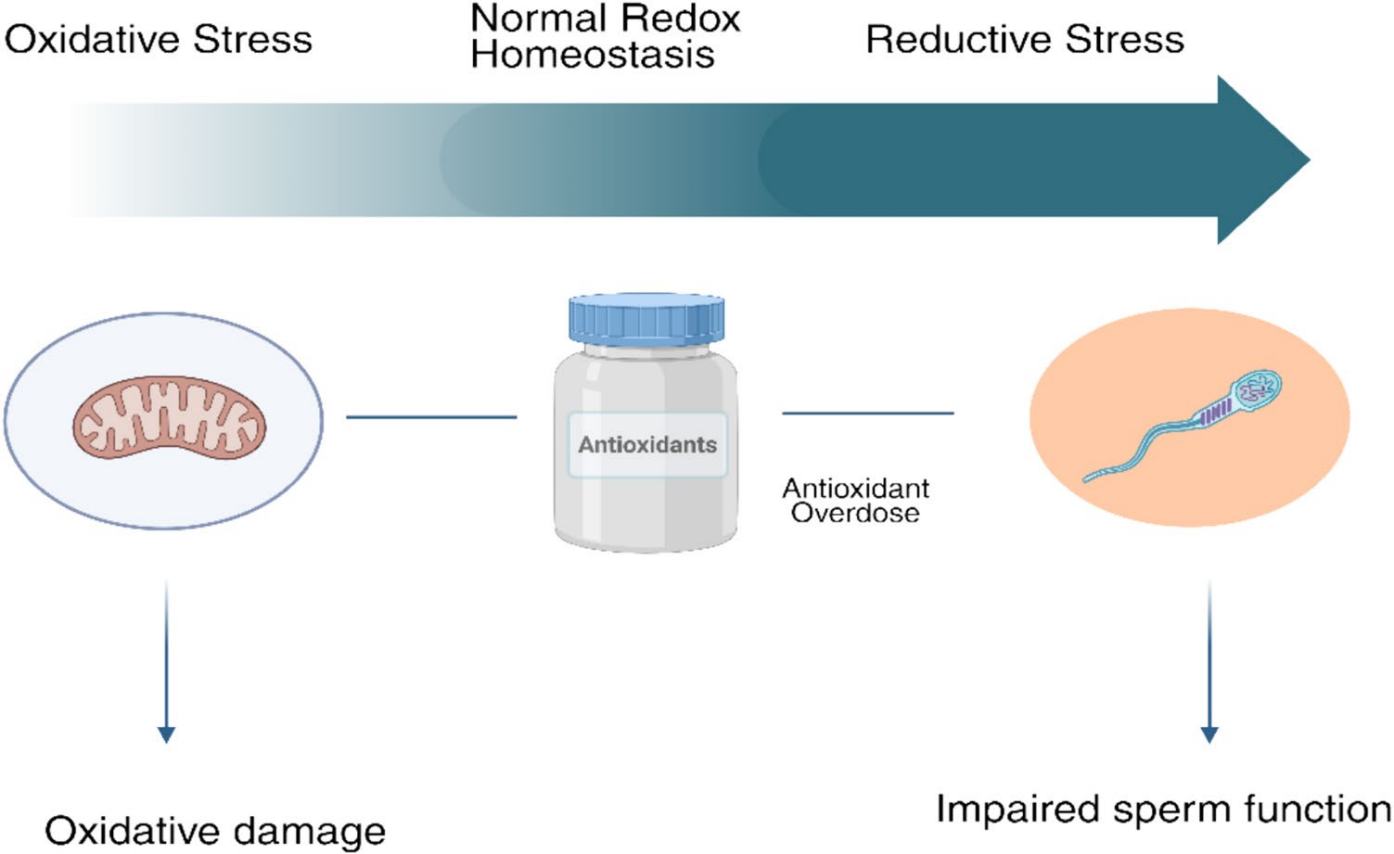
Schematic representation of the impact of antioxidant supplementation on male fertility. Mitochondrial OS contributes to impaired sperm function. Antioxidants counteract OS, potentially improving sperm quality and overall male fertility outcomes over time

Clinicians need to understand that OS and RS represent a continuum, with both extremes potentially disrupting cellular homeostasis [8]. Therefore, attaining redox balance rather than only lowering ROS should be the new therapeutic objective. This has direct implications for the prescription of antioxidant therapy. This has direct implications for the prescription of antioxidant therapy. Rather than relying solely on high-dose or combination regimens, treatment decisions should be guided by a thorough evaluation of the patient's redox status, fertility profile, and response to previous therapies [71]. Figure 3 illustrates a schematic representation of the impact of antioxidant supplementation on male infertility.

In the absence of reliable markers, clinicians should approach extended antioxidant use with caution, particularly when OS has not been established. If clinical improvement is absent, if there is a history of prolonged supplementation, or if semen parameters deteriorate despite treatment, it may be necessary to reassess antioxidant therapy and consider investigating RS [72].

Infertile males frequently self-administer over-the-counter antioxidants without a doctor's supervision, believing that higher dosages produce superior outcomes [59]. Furthermore, these findings carry significant implications for patient counseling. Healthcare providers must emphasize the necessity of treatment under medical supervision and ensure patients are fully aware of the risks linked to excessive antioxidant use [73]. This further underscores the critical need for stringent therapeutic guidelines that integrate redox balance considerations rather than addressing OS in isolation [74].

These clinical observations further underscore the need for coordinated interdisciplinary collaboration among laboratory scientists, andrologists, reproductive endocrinologists, and nutritionists [75]. Integrated care and individualized treatment plans can only achieve the full benefits of antioxidant therapy without sacrificing reproductive outcomes [76].

## Future directions

The expanding understanding of redox biology in male reproduction offers numerous significant avenues for future research and therapeutic development. However, despite advances, the precise role and clinical impact of RS induced by excessive antioxidant supplementation remains insufficiently characterized. A thorough understanding of RS requires further exploration through well-structured, methodologically rigorous clinical studies with adequate sample sizes to address current limitations [16, 26]. Priority should be given to prospective, longitudinal trials that stratify participants by baseline redox status, enabling assessment of dose–response relationships and the definition of safe, effective treatment windows for commonly used antioxidants. Such designs will also help refine the proposed U-shaped effect on sperm function, which currently rests on limited and sometimes inconsistent evidence [38].

An urgent priority is the development of robust, clinically validated biomarkers that can accurately reflect redox status, including both oxidative and reductive imbalances, in real time. Such approaches would allow doctors to track therapy responses, identify early indicators of redox imbalance, and classify patients according to redox profiles [77, 78]. Advances in metabolomics, proteomics, and redox-sensitive imaging may provide mechanisms to achieve this objective, although their clinical applicability requires further validation [79]. In parallel, integrated multi-omics studies could be designed to identify predictive biomarker panels and validate them in targeted patient groups. Future research should also comprehensively evaluate the role of genetic and epigenetic variability in modulating individual responses to antioxidant therapy [80]. Because these genetic variants may impact both treatment efficacy and vulnerability to RS, the presence of polymorphisms in genes involved in redox regulation, mitochondrial function, or antioxidant metabolism supports the application of precision medicine strategies in managing male infertility [10]. However, these findings remain preliminary and highlight the need for more rigorous studies before clinical implementation.

To facilitate clinical translation, there is an immediate need to establish practical frameworks for assessing baseline redox status and monitoring therapeutic responses in patients undergoing antioxidant treatment. Due to the lack of readily accessible or standardized validated redox biomarkers, current clinical practice is mostly on indirect indicators such as patient history and semen analysis. Although more research is needed before they can be routinely used in clinical settings, tests that measure ROS, TAC, and lipid peroxidation products are promising possibilities. To strike a balance between effectiveness and the risk of reducing stress, physicians should use a cautious, step-by-step approach, giving priority to dietary and lifestyle changes first, then closely monitoring antioxidant supplementation. The advancement of customized redox-based treatments will depend on the development of transparent monitoring procedures and decision-making algorithms.

Future research should incorporate larger multidisciplinary viewpoints in addition to molecular and clinical issues. The efficacy of recommended regimens may be determined by behavioral patterns, while psychological factors, such as patient views regarding the "natural" safety of antioxidants, can affect adherence, misuse, or overuse. Reducing inappropriate self-medication could be achieved by public health strategies, such as professional training, awareness campaigns, and regulation of over-the-counter supplements. To ensure viability and adoption in a range of healthcare settings, economic factors, including cost-effectiveness evaluations and fair access to antioxidant therapy, also need to be taken into consideration. Combining insights from medicine, behavioral science, public health, and health economics will help transform redox biology discoveries into effective and sustainable solutions.

Beyond diagnostics and therapeutics, educational initiatives are needed to recalibrate both public and professional perceptions of antioxidants. To effectively address the misuse of antioxidants, redox biology should be incorporated into fertility medicine education, patients must be informed about the potential risks of unsupervised supplementation, and evidence-based prescribing practices should be actively encouraged [81]. Incorporating emerging insights from redox biology into therapeutic practice necessitates coordinated interdisciplinary collaboration. The development of safer, precision-based antioxidant therapies to enhance male fertility while mitigating unintended effects may be substantially accelerated through coordinated interdisciplinary collaboration involving urology, reproductive medicine, pharmacology, and systems biology [82].

## Conclusions

Male infertility has conventionally been managed using antioxidant therapy, particularly when OS is implicated. However, the growing recognition of the potential adverse effects of excessive antioxidant use, notably the induction of RS, underscores the pressing need to reexamine existing treatment protocols. While excessive or long-term use of antioxidants can disturb redox equilibrium, diminish mitochondrial efficiency, and negatively impact spermatogenesis, controlled and moderate supplementation may improve sperm quality [70]. This dilemma underscores the importance of achieving redox balance rather than solely focusing on reducing oxidative markers [83]. The unavailability of standardized clinical protocols and dependable diagnostic tools for redox status assessment renders therapeutic decision-making largely speculative rather than supported by empirical data. Antioxidant therapy remains imprecise and potentially detrimental in the absence of specific biomarkers and individualized treatment strategies [84].

Antioxidant therapy in male infertility should, moving forward, be employed with increased prudence and tailored to the individual patient, supported by comprehensive insights into redox biology [85]. It is essential to ensure that healthcare providers and patients are well informed about the potential risks of improper antioxidant use. Empirical antioxidant use should be supplanted by evidence-based, biomarker-guided therapy to optimize treatment effectiveness and reduce the risk of adverse effects [43].

The antioxidants carnitine, NAC, coenzyme Q10, vitamin C, and vitamin E are most frequently linked to better male reproductive results. When utilized properly, these substances promote mitochondrial and metabolic processes, strengthen redox balance, and shield sperm from oxidative damage. However, abusing them, especially with large dosages or long-term regimens without therapeutic supervision, might result in RS and lower the quality of sperm. A promising substitute with lower risk profiles is provided by antioxidants obtained from natural food sources that are present in the MD. These plant-based substances may be used in conjunction with traditional treatments and have anti-inflammatory and antioxidant properties. Any antioxidant's therapeutic value is contingent upon its type and dosage, as well as the underlying redox state and health status of the individual. Safety and effectiveness should be guaranteed by giving priority to customized biomarker-guided strategies.

## Data Availability

No datasets were generated or analyzed during the current study.
